# State and Municipal Hygiene: With a Plea for a State Board of Health

**Published:** 1893-07

**Authors:** Jas. C. Avary

**Affiliations:** Atlanta, Ga.


### *ORIGINAL COMMUNICATIONS.*

### STATE AND MUNICIPAL HYGIENE: WITH A PLEA FOR A STATE BOARD OF HEALTH.

By JAS. C. AVARY, M. D.,
Atlanta, Ga.

Cholera has been the agent of more good sanitation than any other one cause. The recent simultaneous effort to "clean up" in every town, city or hamlet is the most notable and significant event in modern hygiene. So declares Dr. Lindsley, Secretary of the State Board of Health of our tidy New England sister, Connecticut.

Dr. Joseph F. Edwards, who is Foreign Associate Member of the French Society of Hygiene, and member of the State Board of Health of Pennsylvania, and to whose practical and scientific journal, *Annals of Hygiene,* I am largely indebted for the inspiration and data of this paper, quotes Dr. Lindsley as above, and eloquently adds: "The great apostle of hygiene in this reformation is the cholera. Cholera is a practical preacher, and demonstrates its doctrines by its works. Wherever it finds a people disregarding the lessons it has taught before, it wastes no time in argument, it simply kills them." Europe and America both have had repeated teachings of this sort, and are wise in making the lessons practical. It is to be hoped that they will not be too tardy or too negligent in obeying the behests of this uncompromising, arbitrary and pitiless instructor in nature's laws of health.

Cholera has been very aptly called the great sanitary inspector of nature. When gregarious man too openly defies the laws of health, and wantonly falls into beastly habits of filthiness, the cholera starts on its inspecting tour, and inculcates the laws of healthy living with a decision and emphasis that is best measured by the graves it fills. It is the author of sanitation, and, notwithstanding the destructive energy with which it acts, it has been a benefactor of the human race, having saved more lives by the reforms it has produced than it has destroyed; as Krupp's great guns, by their very deadliness, have made war well nigh impossible.

Since cholera occupies so important a relation to modern hygiene, it might not be amiss or uninteresting to glance at the history of this disease as connected with ourselves. Its first appearance in America was in 1832. In June of that year it appeared in Montreal, Quebec and New York City. It radiated thence along the lines of travel to our other large cities, and did not leave us for a period of four years. In December, 1848, it reappeared in New York, and spread with swiftness and power into the other States. It raged among us during 1849, '50, '51, '52, '53, '54, and not until '55 did our unwelcome guest leave us for the time being.

On November 3d, 1865, the steamship Atalanta, with five hundred German emigrants on board, sailed into New York bay with cholera on board. Immediate quarantine precautions were taken, and we escaped the scourge. But in the following spring, cholera appeared in New York and followed its previous line of travel, abiding with us through 1867. In December, 1872, cholera came to look after us again, and continued through the year 1873. In 1887, and in 1892, cholera knocked at our gates again, but found them barred by the wisdom and firmness of the health officers of New York. We remember that it was hard work for them last summer but they knew what to do, and they did it.

Cholera is no longer a mystery as in the past, but a germ disease, thoroughly understood and capable of easy destruction and control under proper quarantine and sanitary regulations.

Dr. Geo. M. Sternberg, the Deputy Surgeon-General of the United States Army, who is considered a high authority upon this subject, assures us that the cholera germ is one of the most easily destroyed of all known germs. We are assured that a few hours' exposure to dry air is sufficient to destroy it, and that 140° F. may be relied upon to kill it.

The combined experience and aggregate testimony of those who have battled with cholera in hand to hand fight give us the following simple rules as all that is necessary to prevent the spread of cholera among us, even if it should enter our doors.

1. Isolate all drinking water—that is, guard it from all chance of infection by germs. In addition to this precaution, boil all the milk and water that is to be drunk; eat simple and wholesome food, thoroughly cooked.

2. Let all fecal and other animal discharges and all suspicious garbage be destroyed by quick-lime, or consumed by fire.

3. Let all suspected clothing and furnishings be disinfected with dry heat at 212° F., and afterwards with steam, and where it seems advisable see that such things as cannot be thoroughly disinfected are destroyed by fire.

4. Let all cholera corpses be cremated.*

I have given these rules in full, because they are as applicable to every other contagious disease as to cholera, and should be in force all the year round in every city and village and in every rural district.

*In times of great epidemic diseases cremation should be considered an entire necessity.—*Rudolph Virchow.*

What possible good can there be in burning clothes and furniture, if the infected flesh be allowed to remain in existence? In 1868 there was a dreadful epidemic of yellow fever in Lima, Peru, as many as three hundred cases dying daily. Dr. Le Plongeon urged the cremation of the dead. Finally an arrangement was made by which large fires were kept on the trenches filled with corpses. At once the plague abated and soon died out.—*Letter by Alice D. Le Plongeon.*

Dr. E. O. Shakespeare, United States cholera commissioner and late port physician to Philadelphia, who is conceded to know more about the ways of cholera than any one man in the world, assures us that cholera, if it follows its past precedent, will invade us this summer. I am anxious that all boards of health should stand ready girded for combined and intelligent action against its invasion. But I am more anxious that every organization of this kind should awake and keep awake to its duty of destroying those diseases which are endemic among us and which should not be so. While cholera comes among us now and then and kills its thousands, typhoid fever is with us the year round and kills its tens of thousands. Diphtheria, scarlet fever and cholera infantum are all products of filth, and they are never entirely absent from our midst.

The burden of all information on the subject shows that safeguards against visitant and endemic plagues may be summed up in the one word *cleanliness* in living as correlative with *asepsis* in surgery.

Public teaching in school and press, and through boards of health should make a health officer out of every railroad and sleeping car conductor, every school teacher, every preacher, every janitor of a public building; and children in public and private schools should be taught by text-books and practical demonstration the principles of asepsis in living. Our food supply should be guarded by careful and strict legislation—especially that of infants. The Herod of uncleanliness will go on mortifying innocents in our midst as long as our babies die of cholera infantum. The milk supply should be protected in every State and every subdivision of every State by the milk test which shows the food value of the milk to be unimpaired by the addition of water or the subtraction of cream, together with the laboratory test which searches the milk for germs of disease which may be found therein. In addition to these, and more important still, we must have a dairy farm inspection. Every physician, every legislator, every man who is the father of a helpless baby, should take it upon himself to see that we secure by legislative enactment a skillful, efficient and honest inspection of dairy farms and of all places where a cow is milked, or milk is sold on tap or distributed in any way. The inspector's duty should be to see that cows are fed, watered and stabled in a clean and wholesome manner. In case of contagious sickness in the dairyman's family he should isolate the sick and protect the milk or forbid its sale. The State, through her board of health, should distribute from time to time pamphlets of information and instruction to dairymen and to all people engaged in the milk industry. To practice any branch of this industry a license should be required. The State should inform those engaged in the production, distribution or sale of milk, through pamphlets prepared by the State board, of the necessary requirements to secure such license. This license should be forfeited and a heavy fine imposed in consequence of the violation of some of these requirements or laws, such, for instance, as selling the milk of a diseased cow.

That typhoid fever is so often found in mountainous districts is due to the fact that typhoid excreta has been drained in some way into the drinking water. Few doctors now believe that typhoid fever poison enters the system through any other source than by the germs which pass through the mouth and stomach, and generally these germs gain entrance there by getting into the water drank. Of course, they are not born and propagated in pure water, but being in the excrement of some patient suffering with this disease, they are thrown upon the surface of the ground, that washes into the spring or empties into a sink which connects with the well in some way, or is carried off by the sewer to a river miles away to be taken up into the reservoir of some town, and from there imbibed by some innocent municipal father, or member of the board of water commissioners, to propagate its kind in his intestines at the expense of his vitality.

Dr. Welch, of Baltimore, in the January number of *Annals of Hygiene*, says: "The famous Millbank prison of London, was frequently the center of severe epidemics of typhoid fever so long as the water supply was drawn from the Thames river, as it flowed past the prison, but after the supply was taken from an artesian well, only three cases occurred in eighteen years. This change of water supply was carried into effect in the midst of a severe epidemic of cholera, and in six days afterwards that disease suddenly ceased in the prison."

The outbreak of a contagious disease anywhere means that a heavy crime against sanitation has been committed. In every case it means that drinking water has been polluted; it means bad sewerage; sometimes it means polluted food, especially polluted milk supply, and maybe polluted air. Proper sanitation, if persisted in, would render the diseases which I have named things of the past.

The tendency of the people and of the legislative powers to think that boards of health, especially State boards, are organized only for epidemic and visitant scourges, is a matter to be deplored and to be corrected. Side by side with cholera as a reformer stand yellow fever and smallpox, and the lessons which they have taught us ought to be applied with profit to these internal and endemic scourges which I have cited. I believe that the history of each and every board of health would show that such board was the crystallization of public sentiment under the whiplash of an epidemic scourge.

Thus the act of Congress of March 3d, 1879, creating a National Board of Health was the direct offspring of the yellow fever scourge of 1878 and 1879.

In 1891 the records show that the National Board of Health, while not abolished, was not in active operation, Congress having for several years discontinued the appropriation necessary to sustain it.

On March 27th, 1892, there was an act passed for preventing the spread of contagious diseases between the States. This act, in case of emergency demanding prompt action, constituted something like a Board of Health, for the time being, of the President, the Secretary of the Treasury and the Surgeon-General of the Marine Hospital, for the purpose of temporary quarantine between the States.

Thus, the history of our National Board of Health shows plainly that its existence and its every movement is only for quarantine in cases of epidemic scourge; that is, only for the purpose of meeting occasional and not continual evils, outside and not internal foes.

The question of sewerage and of the preservation of the water supply from pollution is one and the same. Sewerage does not annihilate filth or germs of disease ; it transfers them from place to place.

The destruction of germs of disease in night-soil, noxious garbage, etc., before it has a chance to reach and pollute any water supply whatever is the end we should try to bring about. I have been turning this matter over in my brain ever since my first connection with the health department of the city of Atlanta, when my earliest insight began into the real difficulties of the question; the solution I have reached is this:

That in every house in town or country there should be an arrangement by which all animal dejecta and noxious garbage should be destroyed in hermetically sealed furnaces connected with chimneys of said house ; or better still, some chemical agent might be used as the destructive or consuming power at the place of first deposit.

I hope this idea will be proved feasible at an early date. I would be glad for some inventive genius to make his fortune and serve humanity at the same time by constructing an apparatus by which all noxious matter could be destroyed along some such line as I have indicated, at a minimum of private cost, and without public expense. Such destruction of all doubtful or poisonous matter at the point of deposit would solve the vexed questions of sewerage, of unpolluted water supply, and of that monstrous hygienic difficulty : What shall we do with garbage and night-soil ?

How sweet and beautiful and comforting a thought it would be, that man would never be poisoned again at the fountain of life and in the stream of refreshing water, when these questions of water-supply, of sewerage, of night-soil and garbage are solved as they should and can be solved; and they should be solved quickly by science and legislation, hand in hand, so that endemics as well as epidemics will be no more.

Perhaps the greater number of this body is unacquainted with the fact that by an act of the legislature on February 25th, 1875, a State Board of Health was provided for, and that by an amendment the following year each county was given a local board; that fifteen hundred dollars was set apart for the State Board's maintenance, when it was first created; that the two subsequent sessions of the legislature of '76 and '77 also made an appropriation of some insignificant sum. But since the good year of 1877—sixteen years ago—the State Board of Health of Georgia has sweetly slumbered, for want of an appropriation by the legislature.

It gives me pleasure in this connection to refer to the last message of his Excellency, Governor Northen, to the General Assembly, in which he says: "I beg to call the attention of the General Assembly to the necessity for a State Board of Health," and then goes on to cite the several urgent and imperative needs of the State for such a body.

Just previous to my departure for Americus I called upon Governor Northen to know the status of legislation pending at the close of the last session of the legislature, and found him thoroughly alive to the necessity of a State Department of Health and of proper legislation on the subject. His patriotic and humanitarian interest in the matter is clearly shown in the message sent to the legislature, which I here quote:

"It will be remembered that last year there was an epidemic of smallpox on the strip of coast known as Harris's Neck, and the State, powerless to rescue or protect her own citizens, was forced to call on the general government for aid. This aid was granted and the disease was promptly checked and eradicated. It would have been more consistent with the dignity of the State and the duty it owes its citizens if Georgia could have taken steps to protect her own people from the scourge. It would also have been more in harmony with our ideas of local self-control. The threatened visitation of Asiatic cholera to this country reveals to us another peril against which we should provide ample means of defence. We may any year be subjected to the invasion of yellow fever from the Spanish countries to the south of us, or of Asiatic cholera through the northern ports. We are now utterly helpless to deal with these plagues, and should either. menace us we should have to appeal again to the Federal government. Besides these dangers from without, we have many sources of danger within our borders, and an efficient Board of Health would be a constant safeguard to the health of our people and afford a feeling of security against epidemic and disease.

"I most earnestly recommend, therefore, that the General Assembly take such action as will reëstablish the State Board of Health, and provide for its maintenance and efficiency."

He assured me that the State Medical Association would have his hearty coöperation in securing such legislation as would make the State Board of Health a live and active institution of the State.

